# The Effect of Lateral Decubitus Position on Nocturnal Intraocular Pressure over a Habitual 24-Hour Period in Healthy Adults

**DOI:** 10.1371/journal.pone.0113590

**Published:** 2014-11-25

**Authors:** Jie Hao, Yi Zhen, Hao Wang, Diya Yang, Ningli Wang

**Affiliations:** 1 Beijing Tongren Eye Center, Beijing Ophthalmology & Visual Sciences Key Laboratory,Beijing Tongren Hospital, Capital Medical University, Beijing, China; 2 Hebei Eye Hospital, Hebei Key Laboratory of Ophthalmology, Xingtai, China; Wayne State University, United States of America

## Abstract

**Purpose:**

To investigate the effect of lateral decubitus position (LDP) on nocturnal intraocular pressure (IOP) and the effect of LDP on 24-hour habitual IOP pattern in healthy subjects.

**Methods:**

Intraocular pressure was measured every 2-hours using an Accupen Applanation Tonometer (Accutome, USA). During the diurnal period (7:30 am, 9:30 am, 11:30 am, 1:30 pm, 3:30 pm, 5:30 pm, 7:30 pm, and 9:30 pm), IOP was measured in the sitting position under bright light (500–1000 lux) after the subjects had been seated for 5 min. The nocturnal IOP was measured in the supine position, right LDP, and left LDP, with randomized sequences, under dim light (<10 lux) at 11:30 pm, 1:30 am, 3:30 am, and 5:30 am. The subjects were awakened and maintained each position for 5 min before the measurement. The 24-hour habitual IOP patterns were obtained according to the nocturnal position (supine, right LDP and left LDP) for either eye. P<0.05 was considered to be significant.

**Results:**

Nineteen healthy subjects were included with a mean age of 51.3±5.8 years. During the nocturnal period, a significant IOP difference was found between the dependent eye (the eye on the lower side) of LDP and the supine position, but not for all the nocturnal time points. Over a 24-hour period, the effect of LDP on habitual IOP pattern was not statistically significant, although the mean nocturnal IOP and the diurnal-nocturnal IOP change for the right and the left eye in the LDP pattern was slightly higher than that in the sitting-supine pattern.

**Conclusion:**

Significant nocturnal IOP differences existed between the dependent eye and the supine, but did not occur consistently for all time points. Over a 24-hour period, the effect of LDP on habitual IOP pattern was not statistically significant in healthy subjects.

## Introduction

Elevated intraocular pressure (IOP) is a risk factor for the development and progression of glaucoma. It has been shown that IOP can be greatly affected by postural change [1]–[3]. It was previously shown that supine IOP was 1.6–8.6 mmHg higher than the seated IOP in normal individuals [2], [4]–[7], and the elevation was 2.7–9.3 mmHg in individuals with glaucoma [1], [5], [6]. Recent studies have shown that IOP was elevated more in the lateral decubitus position (LDP). Lee et al [8], [9] reported that the IOP of the dependent eye (the eye on the lower side in LDP) was higher than that in the supine position in normal individuals or glaucoma patients. Another study [10] confirmed that the IOP of the dependent eye in LDP was higher than the supine IOP. However, these studies focused on LDP IOP during the diurnal period, and little is known regarding the effect of LDP on IOP during the nocturnal period.

Over a 24-hour period, the human body position is maintained upright during the diurnal period and recumbent (supine, right LDP, or left LDP) during the nocturnal period. Based on the nocturnal recumbent position, there can be three 24-hour habitual IOP patterns for each eye, including sitting-supine, sitting-right LDP, and sitting-left LDP pattern. To date, studies have mainly assessed the effect of supine position on 24-hour habitual IOP pattern (sitting-supine pattern). For example, Liu et al [2], [4] showed that during the nocturnal period, the supine IOP was significantly higher than the seated IOP during the diurnal period for the sitting-supine pattern in healthy individuals. Lee et al [3] demonstrated that the peak-trough difference of the sitting-supine pattern was larger than that of the traditionally recognized sitting-sitting pattern in normal tension glaucoma patients.

De Koninck [11] and Dzvonik [12] indicated that lateral sleep position was preferred with age advance. Kim et al [13] demonstrated that the asymmetry in IOP elevation in LDP was associated with asymmetric visual field loss in glaucoma. However, to our knowledge, there have been no reports assessing the effect of LDP on the 24-hour habitual IOP pattern (sitting-right/left LDP pattern).

This study was to investigate the effect of LDP on nocturnal IOP and the effect of LDP on 24-hour habitual IOP pattern in healthy subjects.

## Methods

The study adhered to the tenets of the Declaration of Helsinki and was approved by the Institutional Review Board of Beijing Tongren Hospital. After explaining the nature and possible consequences of the study, each subject gave their written informed consent for participation in the study.

Nonsmoking paid volunteers were recruited from Handan, Hebei Province in 2013 and underwent detailed ophthalmic examinations, which included noncycloplegic visual acuity, slit lamp biomicroscopy, gonioscopy, IOP (HA-2, Kowa, Tokyo, Japan), central corneal thickness (CCT), and dilated funduscopy. The exclusion criteria were best visual acuity less than 20/40, equivalent spherical refraction greater than 4D, occludable angle [14], IOP ≥21 mmHg, cup/disc ratio ≥0.6, and cup/disc ratio asymmetry ≥0.2, and glaucoma appearing in the fundus (excavation, rim defect, hemorrhage, notching, or visible nerve fiber layer defect). The medication histories of the subjects were recorded. Subjects with history of ocular surgery or diabetes mellitus were excluded.

Before the measurement, the subjects were told to maintain 8-hour sleep for one week and abstain from alcohol and coffee for 3 days. The use of contact lenses was not allowed for 24-hours. The IOP was measured every 2 hours using an Accupen Applanation Tonometer (24–3000, 12K1031, Accutome, USA). Accupen is a handheld electronic applanation tonometry, which is a modified Mackay-Marg tonometer. In our pilot test, for the assessment of accuracy between the Accupen and Goldmann tonometer, 49 eyes were measured by a single, blinded examiner.

The measurement began at 5:30 pm and the right eye was always measured first. One drop of 0.5% proparacaine was used as a local anesthetic. Two measurements were obtained each time. If the difference was more than 2 mmHg, a third measurement was performed, and the two closest values were averaged for the calculation. During the diurnal period (7:30 am, 9:30 am, 11:30 am, 1:30 pm, 3:30 pm, 5:30 pm, 7:30 pm, and 9:30 pm), IOP was measured in the sitting position under bright light (500–1000 lux) after the subjects had been seated for 5 min. Blood pressure and heart rate were subsequently obtained in right arm in the sitting position (OMRON, Japan). The lights were turned off at 11:00 pm. The nocturnal measurements were taken at 11:30 pm, 1:30 am, 3:30 am, and 5:30 am under dim light (<10 lux). The subjects were awakened and asked to maintain each position for 5 min before the measurements were taken. At each time point, the IOP was measured in the supine position, right LDP, and left LDP, in randomized sequences. A soft pillow was placed under the head to maintain the neck in the horizontal position. In the LDP, care was taken not to compress the dependent eye against the pillow. Blood pressure and heart rate were only obtained in right arm in the supine position after the nocturnal IOP measurement. At 7:00 am, the light was turned on and IOP was measured as previously described for the diurnal period. Normal indoor activities were encouraged and napping was allowed.

## Statistics

The IOP of two eyes was analyzed separately without correction by CCT. Wilcoxon signed-rank test was taken to compare the IOP of the dependent or nondependent eye with supine IOP at the nocturnal time points measured. The repeated measures ANOVA test was used to analyze IOPs during the nocturnal period. Three 24-hour habitual IOP patterns were obtained according to the diurnal sitting position and three nocturnal body positions (supine, right LDP, and left LDP), including sitting-supine, sitting-right LDP, and sitting-left LDP pattern. Twelve measurements in each pattern were calculated to obtain the IOP parameters (average, peak, trough, nocturnal, peak-trough difference, and diurnal-nocturnal change) of each individual. Friedman test was used to investigate the differences in IOP parameters among three 24-hour patterns. P values<0.05 were considered to be significant. (SPSS statistical software version 17.0, SPSS Inc., Chicago, IL)

A best-fitting cosine curve [2], [4], [15] was used for the estimation of the 24-hour rhythm in each pattern. The null hypothesis of phase timings distributed randomly in a 24-hour period was tested with a Rayleigh test. A statistically significant difference would indicate a synchronized 24 hour rhythm. In the model, the acrophase (cosinor analysis derived peak time) and amplitude (half distance between the cosine-fit maximum and minimum) represented the phase timing and simulated variation, respectively. Friedman test was used for the comparison in amplitude among three patterns for either eye. P values<0.05 were considered to be significant. (Version7.7.0.471, Math Works. Inc., U.S.)

Mean blood pressure [10] was calculated as the diastolic blood pressure plus one third of the difference between the systolic and the diastolic blood pressures. In the diurnal sitting and nocturnal supine position, ocular perfusion pressures were calculated according to the following formulas proposed by Bill [16]: 1) Sitting ocular perfusion pressure = 95/140×mean blood pressure-IOP; 2) Supine ocular perfusion pressure = 115/130×mean blood pressure-IOP.

## Results

**Figure 1:**
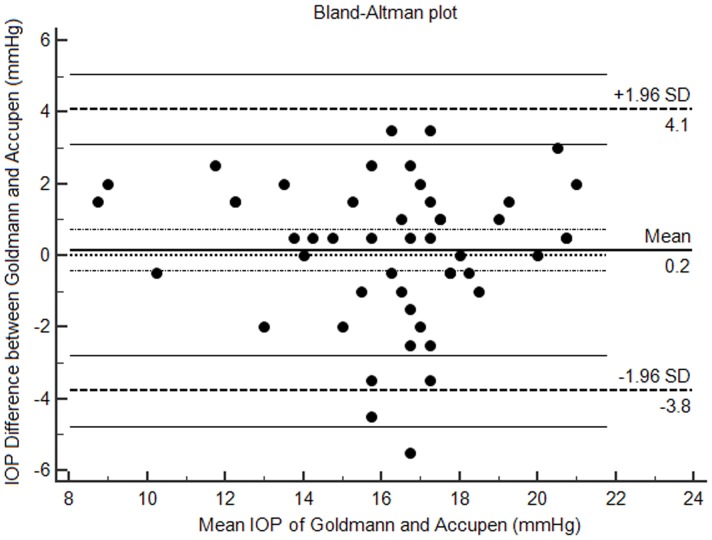
Bland-Altman plot showing the assessment of accuracy between the Accupen and Goldmann tonometers.

The data showed a good agreement between the two tonometers with the intra-class correlation coefficient of 0.873 (p<0.001). Furthermore, the Bland-Altman plot showed that the mean difference (0.2 mmHg) was close to the line of difference of zero, 4.1% (2/49) of dots were outside of the 95% CI (confidence interval) limits of agreement, and the largest difference between the measurements of the two tonometers inside the 95% CI limits of agreement was close to 3.8 mmHg (Figure 1).

Nineteen subjects (16 females and 3 males) were included with a mean age of 51.3±5.8 years (range 40–59 years). With the exception of one subject who had bronchitis and was using ambroxol, all other subjects were healthy, without ocular/systemic comorbidities and medication history. Table 1 shows the baseline characteristics of the subjects. The cup/disc ratio, screening IOP, CCT, and equivalent spherical refraction were not significantly different between the right and left eyes. The blood pressure, heart rate, and ocular perfusion pressure were averaged in diurnal (sitting position) and nocturnal (supine position), separately (Table 2).

**Table 1:** Demographic characteristics.

Demographics	Values	P
**Age (years)**	51.3±5.8 (40–59)	-
**Gender (F/M)**	16/3	-
**Height (cm)**	157.08±5.63 (148.0–168.8)	-
**Weight (kg)**	62.53±11.59 (37.5–89.0)	-
**Screening IOP (mmHg, right eye vs. left eye)**	12.4±2.4 (10.0–20.0) vs. 12.2±2.1 (9.0–17.0)	0.827
**Central Cornea Thickness (µm, right eye vs. left eye)**	520.2±25.8 (478–562) vs. 520.9±24.8 (484–565)	0.739
**Spherical Equivalence (diopter, right eye vs. left eye)**	−0.16±0.80 (−2.50–0.88) vs. −0.24±0.91 (−2.88–1.00)	0.240

Data are expressed as the mean ± SD; Wilcoxon signed-rank test was used for the comparison; P values less than 0.05 were considered significant.

**Table 2:** Systemic parameters.

Parameters	Diurnal	Nocturnal	P
**Mean blood pressure (mmHg)**	89±9 (79–107)	90±11 (78–115)	0.411
**Heart rate (beats/min)**	73.5±6.8 (63–89)	64.9±7.4 (54–86)	0.001
**Mean OPP in right eye (mmHg)**	48.9±6.2 (41.0–62.4)	65.5±10.0 (54.8–87.7)	0.001
**Mean OPP in left eye (mmHg)**	48.4±6.2 (41.0–62.1)	65.7±10.3 (55.3–87.5)	0.001

Data are expressed as the mean ± SD; OPP: ocular perfusion pressures; Wilcoxon signed-rank test was used for the comparison; P values less than 0.05 were considered significant.

At nocturnal measured time points, the effect of LDP on nocturnal IOP is shown in Table 3. It presents the comparison between IOP of LDP (dependent or nondependent eye) and that of the supine position. At 5:30 am, for the right eye, the IOP of the dependent eye was significantly higher than that in supine position (15.7±2.1 mmHg vs. 14.6±2.5 mmHg, p = 0.015). However, for the left eye, the IOP of the dependent eye was lower than that in the supine position (13.9±2.2 mmHg vs. 14.9±2.3 mmHg, p = 0.026). At 11:30 pm, the dependent eye was significantly higher than that in the supine position for the left eye (14.0±2.0 mmHg vs. 12.1±2.8 mmHg, p = 0.005). No significant differences in IOP were found between the dependent eye and the supine at other time points. All the differences between IOP of nondependent eye and that of supine were not significant. Further, using repeated measurement ANOVA for the nocturnal period, there was no interaction identified for the groups (three nocturnal positions) and times (right eye, p = 0.558; left eye, p = 0.292). There was no statistical significance in IOPs among the three nocturnal positions (right eye, p = 0.591; left eye, p = 0.924), but the difference in IOPs at the four time points in each group was statistically significant (right eye, p = 0.003; left eye, p = 0.013).

**Table 3:** Intraocular pressure comparison between the lateral decubitus position and supine position for right/left eye at various nocturnal measured time points.

Time Points	Right Eye (IOP, mmHg)					Left Eye (IOP, mmHg)				
	Supine	Right LDP	Left LDP	P*	P**	Supine	Right LDP	Left LDP	P*	P**
**11:30pm**	13.0±2.5	13.6±3.0	14.2±2.8	0.285	0.136	12.1±2.8	12.9±3.1	14.0±2.0	0.208	**0.005**
**1:30am**	14.2±1.9	14.3±2.3	14.2±1.9	0.932	0.687	13.8±2.8	14.3±3.0	14.3±2.8	0.147	0.541
**3:30am**	13.8±2.6	14.3±2.2	14.2±2.0	0.419	0.296	14.0±3.3	14.2±2.0	13.8±2.1	0.365	0.462
**5:30am**	14.6±2.5	15.7±2.1	14.7±2.0	**0.015**	0.776	14.9±2.3	15.2±2.4	13.9±2.2	0.569	**0.026**

Data are expressed as the mean ± SD; IOP: intraocular pressure; LDP: lateral decubitus position; Wilcoxon signed-rank test was used for the comparisons; P values less than 0.05 were considered significant.

* Intraocular pressure comparison between the right LDP and supine position for right/left eye;

** Intraocular pressure comparison between the left LDP and supine position for right/left eye.

**Figure 2:**
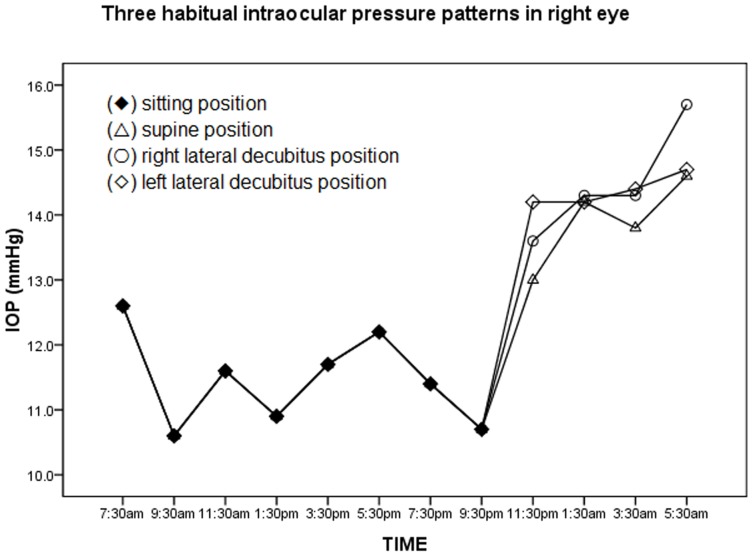
Three 24-hour habitual intraocular pressure (IOP) patterns for the right eye. Three 24-hour habitual IOP patterns were obtained according to the diurnal sitting position (⧫) and nocturnal body positions, including supine position (△), right lateral decubitus position (○), and left lateral decubitus position (◊).

**Figure 3:**
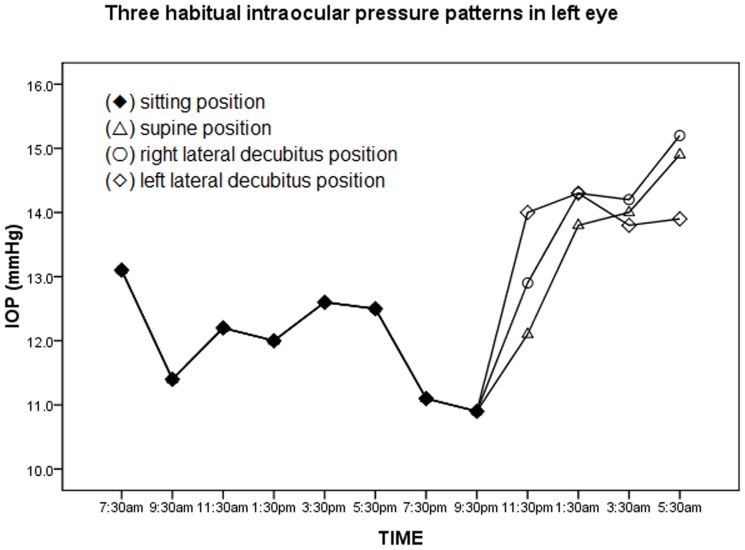
Three 24-hour habitual intraocular pressure (IOP) patterns for the left eye. Three 24-hour habitual IOP patterns were obtained according to the diurnal sitting position (⧫) and nocturnal body positions, including supine position (△), right lateral decubitus position (○), and left lateral decubitus position (◊).

Over a 24-hour period, three habitual IOP patterns are observed, as shown in Figure 2 and Figure 3. The mean nocturnal IOP was significantly higher than the diurnal in each pattern for either eye (p<0.001). Table 4 shows the IOP parameters among three circadian habitual IOP patterns. The differences in IOP parameters were not statistically significant among the three patterns for either eye, especially the mean nocturnal IOP or the diurnal-nocturnal IOP change. In the right eye, the mean nocturnal IOP in the sitting-right/left LDP patterns (14.5±1.8 mmHg/14.4±1.7 mmHg) were slightly higher than the sitting-supine pattern (13.9±1.8 mmHg, p = 0.123); the diurnal-nocturnal IOP change in the sitting-right/left LDP patterns (3.0±1.0 mmHg/2.9±1.4 mmHg) were also slightly higher than the sitting-supine pattern (2.5±1.3 mmHg, p = 0.520). In the left eye, the mean nocturnal IOP in the sitting- right/left LDP patterns (14.1±1.8 mmHg/14.0±1.6 mmHg) were higher than the sitting-supine pattern (13.7±2.0 mmHg, p = 0.123). Additionally, the change in diurnal-nocturnal IOP in the sitting-right/left LDP patterns (2.2±1.6 mmHg/2.0±1.9 mmHg) were also higher than the sitting-supine pattern (1.7±1.4 mmHg, p = 0.520).

**Table 4:** Intraocular pressure parameters for the three circadian patterns in the right/left eye.

IOP Parameters (mmHg)	Right Eye				Left Eye			
	Sitting-supine pattern	Sitting-right LDP pattern	Sitting-left LDP pattern	P	Sitting-supine pattern	Sitting-right LDP pattern	Sitting-left LDP pattern	P
**Peak**	16.0±2.0	16.4±2.0	16.2±1.7	0.174	16.6±2.7	16.8±2.3	16.4±2.3	0.361
**Trough**	8.8±2.1	8.9±2.1	8.9±2.1	0.368	9.2±2.0	9.2±1.9	9.0±1.9	0.522
**Peak-Trough**	7.2±2.0	7.5±2.0	7.4±2.0	0.269	7.5±2.0	7.6±1.8	7.4±2.0	0.647
**Average**	12.3±1.7	12.5±1.8	12.4±1.7	0.092	12.6±1.8	12.7±1.7	12.7±1.5	0.520
**Diurnal**	11.5±1.8	11.5±1.8	11.5±1.8	-	12.0±1.8	12.0±1.8	12.0±1.8	-
**Nocturnal**	13.9±1.8	14.5±1.8	14.4±1.7	0.123	13.7±2.0	14.1±1.8	14.0±1.6	0.520
**Diurnal-Nocturnal**	2.5±1.3	3.0±1.0	2.9±1.4	0.123	1.7±1.4	2.2±1.6	2.0±1.9	0.520

Data are expressed as the mean ± SD; LDP: lateral decubitus position; Friedman test was used for the comparisons among three circadian patterns; P values less than 0.05 were considered significant.

**Figure 4:**
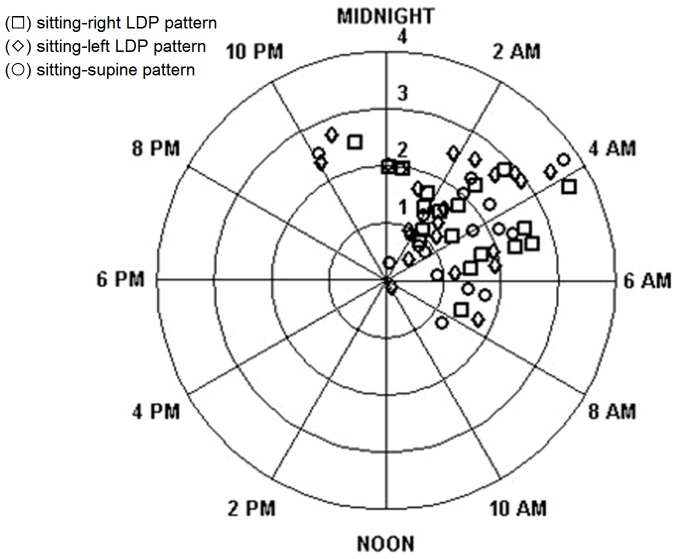
Estimation of 24-hour intraocular pressure (IOP) rhythm in each pattern for the right eye. The position of the acrophase around the circle shows its timing, and the radial distance from the center shows the amplitude of the IOP rhythm. The null hypothesis of a random circular distribution of acrophases was rejected by the Rayleigh test for each age group (p<0.001). The square (□) represents the sitting-right LDP (LDP, lateral decubitus position) pattern; the rhombus (◊) represents the sitting-left LDP pattern; the dot (○) represents the sitting-supine pattern.

**Figure 5:**
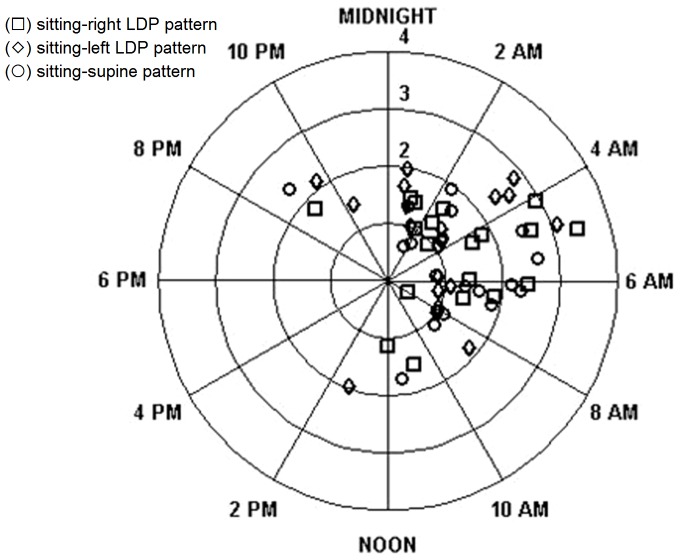
Estimation of 24-hour intraocular pressure (IOP) rhythm in each pattern for the left eye. The position of the acrophase around the circle shows its timing, and the radial distance from the center shows the amplitude of the IOP rhythm. The null hypothesis of a random circular distribution of acrophases was rejected by the Rayleigh test for each age group (p<0.001). The square (□) represents the sitting-right LDP (LDP, lateral decubitus position) pattern; the rhombus (◊) represents the sitting-left LDP pattern; the dot (○) represents the sitting-supine pattern.

With the exception of the sitting-supine pattern, significant rhythms were found in sitting-right/left patterns for either eye (p<0.001). For the right eye, the acrophases were 3:12±1.11 am, 3:15±1.12 am, and 2:53±1.53 am in the sitting-supine, sitting-right LDP, and sitting-left LDP pattern. For the left eye, the corresponding values were 5:09±1.03 am, 4:31±1.10 am, and 3:42±1.55 am, respectively. The amplitudes in the right eye were 1.4±0.2 mmHg, 1.7±0.2 mmHg, and 1.6±0.2 mmHg for the sitting-supine, sitting-right LDP, and sitting-left LDP pattern. For the left eye, the values were 1.1±0.2 mmHg, 1.2±0.2 mmHg, and 1.1±0.2 mmHg, respectively. No significant differences were found in amplitude among three patterns for either eye (Figure 4 and Figure 5).

## Discussion

This study is the first to report the effect of LDP on nocturnal IOP over a 24-hour period in healthy subjects. At nocturnal time points, a significant IOP difference existed between the dependent eye and the supine, but not for all time points. Over the habitual 24-hour period, no statistical IOP differences were found among the three habitual patterns for either eye, although the mean nocturnal IOP or diurnal-nocturnal IOP change in the LDP pattern was slightly higher than that in the sitting-supine pattern. Synchronized 24-hour rhythms were found in the sitting-right/left LDP patterns.

The effect of LDP on IOP has usually been investigated in single time point under light conditions. The present study focused on the postural change at different nocturnal time points. First, our results found that significant IOP difference existed between the dependent eye and the supine for some nocturnal time points, but not for all time points. Most LDP studies [8]–[10] reported that the IOP of dependent eye was higher than the supine IOP. However, previous LDP studies were carried out at single time point during the diurnal period. To our knowledge, the present study was the first to report LDP IOP with different nocturnal time points. At some time points, we did not find this difference. The IOP of dependent eye was even lower than the supine at 5:30 am for the left eye. The reason for the discrepancy is unclear. Repeated measurement ANOVA analysis showed that the main effect of nocturnal time was significant, indicating that IOP varied through the nocturnal period. It may partly explain the differences at the four nocturnal time points.

Second, the difference in the right dependent eye compared with left dependent eye may be caused by right-left differences in the cardiovascular system. Malihi et al [17] showed that IOP in the right dependent eye was significantly higher than the supine, but not in the left dependent eye. They speculated that the heart was left-sided, which may cause cardiovascular changes in LDP. This would affect episcleral venous pressure and IOP, which may result in different responses during right/left LDP. Further studies on this discrepancy are warranted.

Third, no significant differences were found between the nondependent eye and the supine at the nocturnal measured time points for either eye. In the present study, 5 min was taken as the interval for maintenance of each position, which has previously been proven effective [8], [10], [17]. Lee et al [8] also found that the IOP difference between the nondependent eyes and supine was not statistically significant at 5 min, and even at 30 min after LDP. Another study [10] showed that the IOP was higher in the dependent eye of the LDP compared with the ipsilateral eye of the supine position. However, they also showed such a difference was not found in the nondependent eye of the lateral and supine position.

Over a 24-hour habitual period, the IOP parameters in the sitting-right/left LDP patterns were not statistically significantly higher than the sitting-supine pattern for either eye. The results were consistent with the repeated measurement ANOVA analysis, which showed that there was no statistically significant difference in IOPs among three nocturnal positions. This may be attributed to the inconsistent IOP difference at the nocturnal measured time. In addition, measurements in the recumbent position were limited for the three patterns. To simulate the habitual position through the 24-hour period, the recumbent position was only adopted during the nocturnal period. Moreover, in order to avoid disturbing the circadian rhythm, there were only four nocturnal measurements. Hence, the effect of different postures on circadian pattern may not reflect the real 24-hour habitual pattern.

Significant rhythm with nocturnal phase timing (acrophase) was found in the three circadian habitual patterns. It indicates that IOP frequently appears higher in a recumbent position. The IOP elevation in the recumbent position may be due to the changes in episcleral venous pressure [18], choroidal vascular volume [19], and hydrostatic effects [17]. Compared with the right eye, the acrophase in the left eye was delayed and distributed in more areas. It is known that the association between the two eyes was only moderate [20], but the reasons of the acrophase differences are unknown. Whether or not the differences between two eyes are clinically significant remain to be evaluated.

Limitations in our study should be noted. First, the tonometer in our study was Accupen, which was not compared with the Goldmann tonometer in the supine position. The Goldmann tonometer is still regarded as the gold standard for IOP measurements. However, the necessary upright position precludes its comparison in different body position. Second, the limited nocturnal measurement may preclude the effect of the recumbent position on circadian patterns. Although IOP monitoring with a wireless ocular telemetry sensor has been used for continuous records, its value is not obtained in mmHg, and the relationship of these measurements to IOP is not known. Last, other systemic parameters, such as blood pressure and heart rate, may have effects on postural IOP change, and were not measured in the lateral position in the present study. Further studies on the effect of systemic parameters on postural IOP change need to be investigated.

In conclusion, IOP difference existed between the dependent eye and the supine, but not for all the nocturnal time points. Over a habitual 24-hour period, the effect of LDP on circadian habitual IOP pattern was not statistically significant in healthy subjects. Further studies to investigate the effect of LDP on nocturnal IOP during a habitual 24-hour period are warranted in glaucoma patients.
